# Automated detection and segmentation of thoracic lymph nodes from CT using 3D foveal fully convolutional neural networks

**DOI:** 10.1186/s12880-021-00599-z

**Published:** 2021-04-13

**Authors:** Andra-Iza Iuga, Heike Carolus, Anna J. Höink, Tom Brosch, Tobias Klinder, David Maintz, Thorsten Persigehl, Bettina Baeßler, Michael Püsken

**Affiliations:** 1 Institute of Diagnostic and Interventional Radiology, Medical Faculty and University Hospital Cologne, University of Cologne, Kerpener Str. 62, 50937 Cologne, Germany; 2 Philips Research, Röntgenstraße 24, 22335 Hamburg, Germany; 3 Institute of Diagnostic and Interventional Radiology, University Hospital Zürich, Zürich, Switzerland

**Keywords:** Deep learning, Artificial intelligence, Lymph nodes, Computed tomography, Staging

## Abstract

**Background:**

In oncology, the correct determination of nodal metastatic disease is essential for patient management, as patient treatment and prognosis are closely linked to the stage of the disease. The aim of the study was to develop a tool for automatic 3D detection and segmentation of lymph nodes (LNs) in computed tomography (CT) scans of the thorax using a fully convolutional neural network based on 3D foveal patches.

**Methods:**

The training dataset was collected from the Computed Tomography Lymph Nodes Collection of the Cancer Imaging Archive, containing 89 contrast-enhanced CT scans of the thorax. A total number of 4275 LNs was segmented semi-automatically by a radiologist, assessing the entire 3D volume of the LNs. Using this data, a fully convolutional neuronal network based on 3D foveal patches was trained with fourfold cross-validation. Testing was performed on an unseen dataset containing 15 contrast-enhanced CT scans of patients who were referred upon suspicion or for staging of bronchial carcinoma.

**Results:**

The algorithm achieved a good overall performance with a total detection rate of 76.9% for enlarged LNs during fourfold cross-validation in the training dataset with 10.3 false-positives per volume and of 69.9% in the unseen testing dataset. In the training dataset a better detection rate was observed for enlarged LNs compared to smaller LNs, the detection rate for LNs with a short-axis diameter (SAD) ≥ 20 mm and SAD 5–10 mm being 91.6% and 62.2% (*p* < 0.001), respectively. Best detection rates were obtained for LNs located in Level 4R (83.6%) and Level 7 (80.4%).

**Conclusions:**

The proposed 3D deep learning approach achieves an overall good performance in the automatic detection and segmentation of thoracic LNs and shows reasonable generalizability, yielding the potential to facilitate detection during routine clinical work and to enable radiomics research without observer-bias.

## Background

The correct determination of nodal metastatic disease is imperative for patient management in oncology, since the patients’ treatment and prognosis are inherently linked to the stage of disease [1]. For nodal disease staging of solid tumors, unidimensional measurements of lymph node (LN) short-axis diameters (SAD) are routinely performed during tumor staging and re-staging imaging examinations and evaluated according to different standardized diagnostic criteria such as the Response Evaluation Criteria in Solid Tumors (RECIST) [2]. For lymphomas, a different set of standardized diagnostic criteria such as the Response Evaluation Criteria in Lymphoma (RECIL) [3] or the Lugano Criteria [4] have been suggested, using bi- instead of unidimensional LN measurements.

Although it is commonly accepted that larger LNs have a higher probability of being malignant as compared to smaller LNs, previous work has shown that enlargement of LNs alone is not the most reliable predictive factor for malignancy with only 62% sensitivity and specificity being demonstrated for predicting LN metastasis in patients with non-small cell lung cancer when using the proposed 10 mm cut-off [5]. Consequently, small LNs potentially harboring micrometastases should be taken into consideration for improved diagnostic accuracy during disease staging [6–8]. Unfortunately, no imaging technique (including, e.g., functional techniques such as diffusion-weighted magnetic resonance imaging) so far has been demonstrated to be capable of reliably detecting LN micrometastases [9–11].

Radiomics is a promising novel strategy for predicting LN dignity from images. Radiomic models thereby are built using e.g., machine learning algorithms based on a large set of quantitative features, which are mathematically or statistically derived from medical images [12–14]. For extraction of radiomic features and detection of LN macro- as well as micrometastases, a reliable and correct detection and whole-volume segmentation of small as well as large LNs is needed. Manual or even semi-automated segmentation of LNs is extremely time-consuming and LN detection strongly depends on the radiologist’s experience, thus currently hampering the translation of a radiomics-based decision support to clinical routine. Consequently, fully automated approaches are urgently needed for a fast and robust detection and segmentation of LNs.

Recent developments in deep learning (DL) have shown promising results in areas relying on imaging data, especially in radiology [15, 16] and cancer imaging [17, 18]. While requiring little human input, DL algorithms significantly outperform existing detection and segmentation methods [19], thus offering automated quantification and selection of the most robust features, including a proper 3D assessment of lesions. Moreover, previous work showed that 3D DL architectures were successful in learning high-level tumor appearance features outperforming 2D models [20]. In cancer imaging, the use of artificial intelligence (AI) has shown a great utility not only in the (semi)automatic tumor detection, but also in tumor characterization, and treatment follow-up. In clinical practice, AI has been lately used in digital pathology, in imaging of the brain for the detection of metastasis and in imaging of the chest, for the early detection of breast carcinoma [17, 18].

Regarding LNs a wide range of 2D approaches [21, 22] have been proposed so far for detection and segmentation, where LNs were segmented using unidimensional measurements consisting of the determination of the SAD of the target lesions. However, a unidimensional approach can underestimate the size as well as the growth of LNs, especially when considering enlarged LNs. Consequently, correct segmentation of LNs considering the whole volume of the lesion is of ultimate importance for proper diagnosis and follow-up.

Thus, the aim of the study was to develop a tool for automatic 3D LN detection and segmentation in computed tomography (CT) scans using a fully convolutional neural network based on 3D foveal patches.

## Methods

### Description of the training and validation dataset

For the training and validation dataset, images were obtained from the CT Lymph Nodes Collection of the Cancer Imaging Archive [22]. The dataset can be accessed and downloaded at https://wiki.cancerimagingarchive.net/display/Public/CT+Lymph+Nodes. The dataset was made available to allow for a direct comparison to other detection methods in order to advance the state of the art and to encourage development and improvement of computer-aided detection methods. The dataset contained contrast-enhanced CT images of 90 patients from different scanners with an in-slice resolution between 0.63 and 0.98 mm and a slice thickness ranging from 1 to 5 mm (88 CT scans with a slice thickness of 1 or 1.5 mm and 2 CT scans with a slice thickness of 5 mm). To the best of our knowledge, there is no information available regarding patients' disease or further demographic information. The included CT scans showed normal-sized thoracic LNs (SAD < 10 mm) as well as lymphadenopathy (SAD ≥ 10 mm). The datasets included also CT scans containing mediastinal bulky disease and bulky axillary lymphadenopathy. In order to allow better comparison to clinical routine with usually heterogeneous datasets, these were not excluded from network training. One case was excluded from our study since it did not contain the complete scan of the thorax.

For this dataset Institutional Review Board approval was not required because it is a publicly available dataset.

### Description of the testing dataset

Further, a second unseen dataset was collected for independent testing. Similar to the training and validation dataset, the testing dataset consisted of contrast-enhanced CT scans (n = 15). The patients (8 male, 7 female; mean age 68 ± 16.6 years) were referred upon suspicion or for staging of bronchial carcinoma from March 2016 to November 2017 (Table 1). All examinations were performed on a 128-slice PET/CT-system (Siemens Biograph mCT Flow 128 Edge, Siemens Medical). Patients were scanned supine in cranio-caudal direction during inspirational breath-hold after intravenous injection of 120 ml contrast medium (Accupaque 350, GE Healthcare) with an injection rate of 2.5 ml/s and a delay of 60 s. The following scan parameters were used: collimation 128 × 0.6 mm, rotation time 0.5 s, pitch 0.6. All axial images were reconstructed with a slice thickness of 2 mm. Similar to the training and validation dataset, the testing dataset included CT scans, that showed both normal-sized thoracic LNs and lymphadenopathy.

**Table 1:** Demographic details (age and sex) for all patients included in the test dataset

	Age	Sex
1	33	Female
2	64	Female
3	79	Male
4	69	Female
5	79	Male
6	63	Male
7	75	Male
8	68	Male
9	74	Female
10	47	Male
11	33	Female
12	69	Female
13	72	Male
14	33	Female
15	67	Male

Ethical approval was waived due to the retrospective design of the study based on preexisting images (Ethics Committee of the Faculty of Medicine, University of Cologne, reference number 19-1390/ 07.08.2019).

### Lymph node segmentation

#### Training and validation dataset

**Fig. 1:**
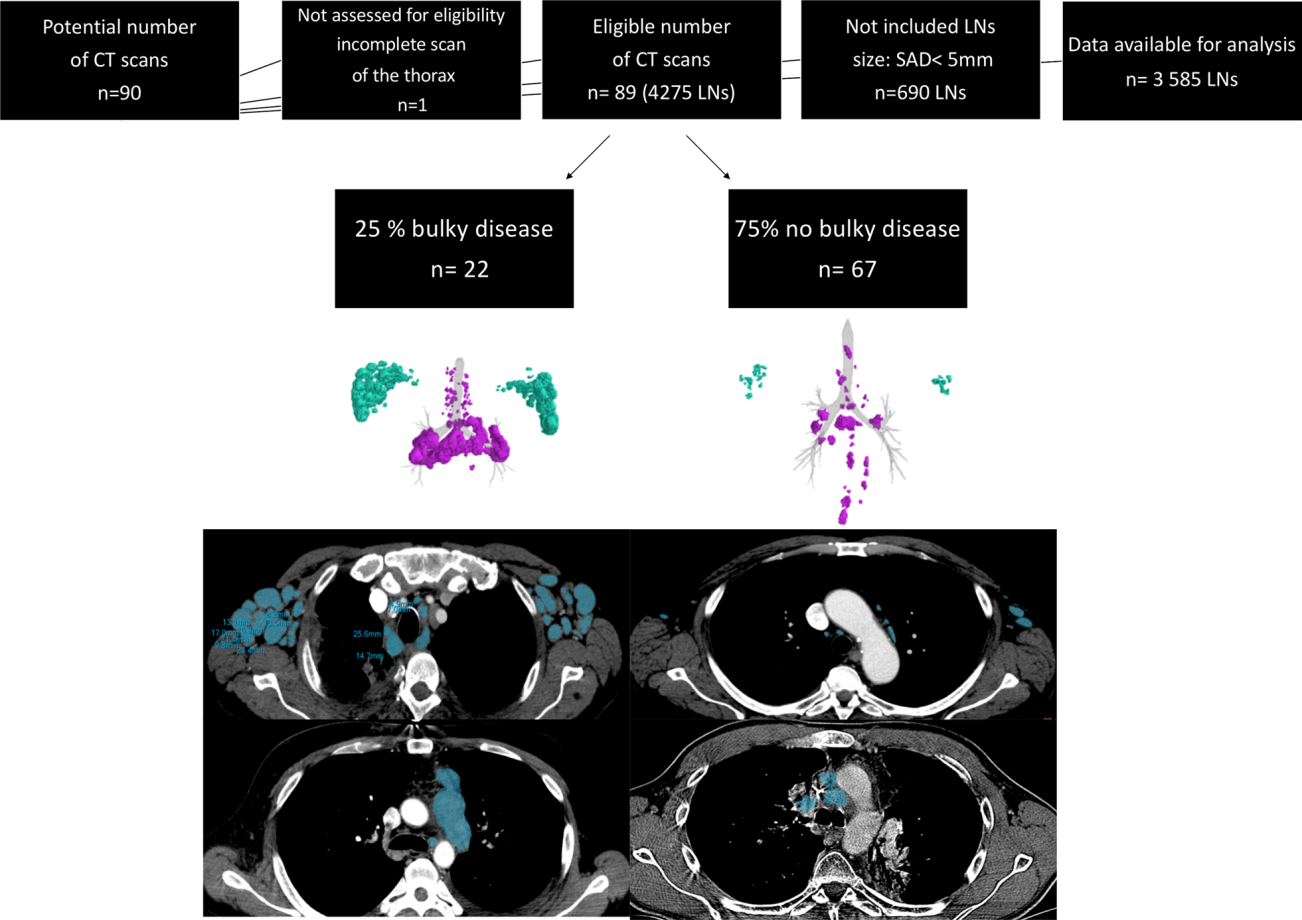
Flow-chart showing data in- and exclusion together with segmentation for network training. From a total number of 90 contrast-enhanced CT scans contained in the publicly available dataset 1 CT scan was excluded because it did not contain the complete scan of the thorax. Further, a total of 690 LNs were excluded because of an SAD < 5 mm. CT scans containing mediastinal bulky disease and bulky axillary lymphadenopathy were not excluded. Top left image—exemplary segmentation of bulky axillary lymphadenopathy; top right image—exemplary segmentation of normal axillary LNs; Bottom left image—exemplary segmentation of bulky mediastinal lymphadenopathy; Bottom right image—exemplary segmentation of enlarged LNs. Considerable differences in image quality of the different CT scans was noted as exemplarily shown in the bottom right image. *CT* computed tomography, *LNs* lymph nodes, *SAD* short-axis diameter

A radiologist (blinded; more than 4 years of experience in thoracic imaging) segmented all LNs of the training and validation dataset with an SAD of at least 5 mm in the mediastinal, hilar and axillary regions using the semi-automatic 3D Multi-Modal Tumor Tracking tool of a commercially available software platform (IntelliSpace Portal, Version 11.0, Philips Healthcare). In case of unclear LNs or findings CT images were discussed with an experienced radiologist with more than 15 years clinical experience and focus in oncological imaging. The training and validation dataset consisted of 4275 LNs, with an average of 48 LNs per patient. When considering the location of the LNs the 4275 LNs included 2272 axillary and 2003 mediastinal/hilar LNs. The LNs had an SAD of 1.3–67.6 mm. A total number of 814 enlarged (SAD > 10 mm) LNs was segmented. The segmentation of the LNs took approximately between 45 and 120 min per dataset. LNs with an SAD < 5 mm that had been mistakenly annotated (n = 690) were not included in the evaluation. Figure 1 shows the process of data collection and LN segmentation.

For data evaluation segmented LNs were divided into 3 groups based on their SAD: 5–10 mm (2523 LNs); 10–20 mm (954 LNs); and > 20 mm (107 LNs).

Furthermore, based on their localization all segmented LNs were divided into axillary (right, left) and mediastinal (including hilar LNs). The mediastinal LNs were further divided in 11 groups depending on their location corresponding levels (levels 1–11), based on the Mountain–Dresler modification of the American Thoracic Society LN map [23]. The side (right respectively left) was considered for level 1, level 2, level 4, level 10 and level 11.

#### Testing dataset

In the testing dataset, a total of 113 LNs were segmented, with an average of 7.5 LNs per patient. The segmented LNs included both axillary and mediastinal/hilar LNs. In this dataset, nevertheless, because of time constraints only LNs with an SAD > 10 mm were segmented.

### Network architecture

A 3D fully convolutional neural network (u-net) was trained on the training dataset, which obtains as input the original 3D images and the corresponding label masks of the segmented LNs. The output of the network was a probability map, showing the probability of each voxel belonging to a mediastinal or axillary LN. This probability map was assessed with a fixed threshold of 0.4 to obtain the final segmentation result. The threshold value was optimized on the training images to yield the best Dice value over all training samples. Finally, a connected component analysis is applied to obtain the individual predicted LNs.

The segmentation network was trained on the 3D images. The used network architecture, named foveal neural network (f-net) [24] is inspired by the human eye and the distribution of the photoreceptor cells, which have the highest resolution at the fovea centralis. A f-net architecture has been used because this architecture combines information of different resolution levels. On the one hand, LNs were analyzed in high resolution to enable feature learning (texture, shape and size). On the other hand, neighboring anatomy was analyzed in low resolution.

**Fig. 2:**
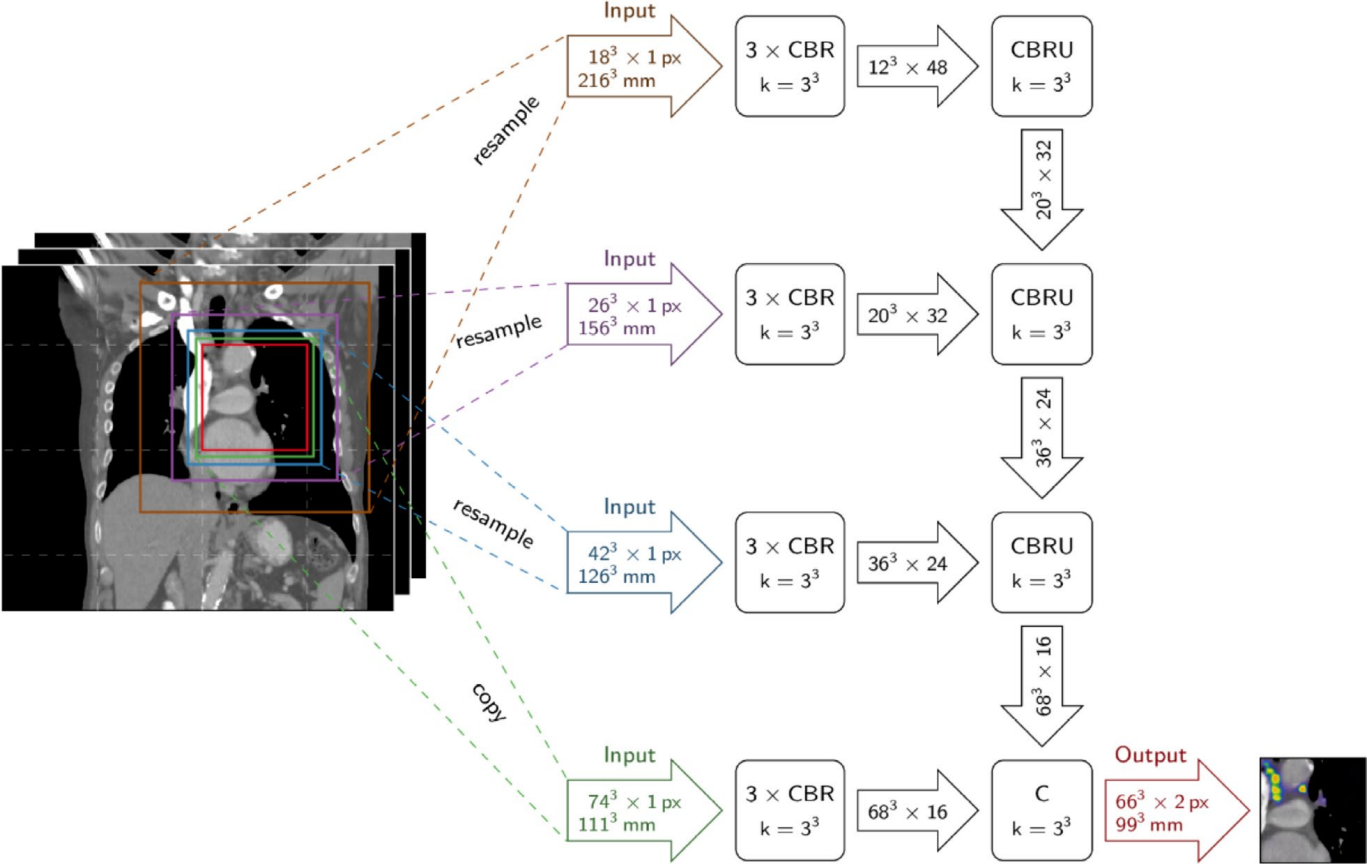
Sketch of the network architecture. A 3D fully convolutional foveal neural network was trained. The network architecture is inspired by the human eye and the distribution of the photoreceptor cells, which have the highest resolution at the fovea centralis. The network consists of several blocks of convolutional layers, batch normalization and the rectified linear activation function (CBR), which extract features at different resolution levels. CBR blocks are followed by upsampling layers (CBRU) to match the resolution of the other levels

As previously mentioned, the network considers image patches at multiple resolution scales in order to arrive at the final prediction, combining local information gained from high resolutions with context from lower resolutions. Unlike u-nets [25], which receive a single scale input image and create the coarser resolution scales by downsampling within the network, f-net directly receives the input as a multiscale pyramid of image patches. Here, an architecture with four resolution levels was used. Accordingly, each input sample to the network consisted of four image patches at the same position but downscaled for the lower resolution levels. The input to each resolution level is processed in a feature extraction pathway. Thus, the number of feature extraction pathways is equivalent to the number of resolution levels in the network. Each feature extraction pathway comprises three successive blocks of valid convolution with a kernel size of 3, batch-normalization, and rectified linear activation function, so called convolutional layer (Conv-L), batch normalization layer (BN-L), and ReLU layer (ReLU-L) (CBR) blocks. The outputs of the feature extraction levels are combined in a feature integration pathway through an additional CBR block followed by upsampling of the lower resolution outputs. Finally, a channel-wise softmax layer is applied to acquire pseudo class probabilities for the LN labels. In addition, f-net was chosen because its architecture requires less memory and runtime compared to u-net [25]. Figure 2 shows an overview of the network architecture.

### Training and validation setup

Training was performed using Microsoft Cognitive Toolkit CNTK with a Python interface (Hardware: 2.40 GHz processor with 2 × NVIDIA GTX 1080ti with 11 GB graphics memory). The images were pre-processed by resampling them to a fixed isotropic sampling grid with a spacing of 1.5 mm, this increases the speed of the network training and deployment while preserving sufficient image detail. Used matrix size was standard (512 × 512) and data pixel size was 1 mm isotropic.

To enhance the soft-tissue contrast of the LNs, only the gray-value window 750/70 Hounsfield Units (HU) was considered and gray-values outside this range were clipped to the upper or lower limit. This gray-value window was determined automatically on the training data by computing the mean and standard-deviation of all voxels labeled as LN and their direct neighborhood. No further pre-processing was performed.

Training was performed based on patches (hereby, it was ensured that at least 30% of the patches contain LN voxels), which were drawn randomly from the images. As data augmentation, random scaling and rotation of the patches was applied on-the-fly with a maximal scaling factor of 1.1 and a maximal rotation of 7°. The usage of stronger augmentation with regard to rotation and scaling showed a decline in performance and was therefore abandoned. Individual LNs were not manipulated during data augmentation and therefore the total number of LNs remained unchanged. Test-time augmentation has not been performed.

The cross-entropy function was chosen for the optimization of the network since it showed good performance on many tasks [12, 13, 26].The network was trained for 1000 epochs with a minibatch size of 8 and the AdaDelta optimizer.

The models were trained using fourfold cross-validation on the training data with the dataset being randomly split into four groups (i.e., training was performed on 3 of the groups while the remaining group was used for validation.). The validation was used to explore performance of the network architecture and training setup with regard to number of resolution levels in the network, optimizer, augmentation and patch sampling strategy. In the following we present the results of the best training experiment. A full ablation study is beyond the scope of this paper and will be addressed in future work.

### Testing setup

Finally, the model trained on the complete training dataset was tested on the previously unseen, in-house derived testing dataset.

### Evaluation criteria

The performance of the network is assessed by looking at the individual LNs. For the ground-truth the single nodes are available from the annotation process. For the predicted LNs, a connected component analysis of the predicted segmentation mask is performed.

One performance metric is the detection rate, which is the number of detected LNs divided by the total number of LNs. A LN is thereby counted as detected if there was at least one voxel overlap with the segmentation mask predicted by the network.

The second performance metric is the number of false positives (FP) per volume. Here, a connected component in the predicted segmentation mask without overlap to a ground truth is counted as FP.

This rather loose criterion was chosen instead of stricter measures, e.g., larger overlap thresholds between ground truth and predicted segmentation, as one particular challenge in LN assessment is that differentiation of individual nodes is often not possible when adjacent nodes merge into clusters due to pathology. Obviously, it can occur that a ground truth segmentation is 'detected' by multiple predicted segmentations and similarly that a predicted segmentation overlaps with multiple ground-truth segmentations. This criterion appears to be current state-of-the-art and has been used in previous work [22].

In addition, the segmentation quality is assessed on a voxel level per image for the detected LNs. To this end, all missed LNs are removed from the ground-truth mask and all FP are removed from the predicted segmentation mask. From the resulting masks Dice, true-positive rate and positive predictive value are computed.

### Statistical analysis

Statistical analysis was performed in the open-source statistics package R version 3.3.1 for Windows (R: A language and environment for statistical computing, R Core Team, R Foundation for Statistical Computing. ISBN 3-900051-07-0, 2019, URL http://R-project.org/). After assessing normal distribution of the data, a two-sided unpaired t-test was applied to determine the differences in means of the detection rates considering both size and location of the LNs. Statistical significance was defined as *p* ≤ 0.05. To get an impression of the variability of the observed detection rates and to confirm the robustness of the results, bootstrapping analysis was performed (with replacement using 100% of the sample size with the number of simulations N = 10.000).

## Results

Calculation of the LN probability maps took about 24 s per dataset on a graphics processing unit (GPU), while training took 120–180 min.

Bootstrap analysis was performed and confirmed the robustness of the results. The empirical distribution of the detection rate showed a standard deviation of 1.7%.

### Network performance: validation dataset

#### Segmentation accuracy

**Fig. 3:**
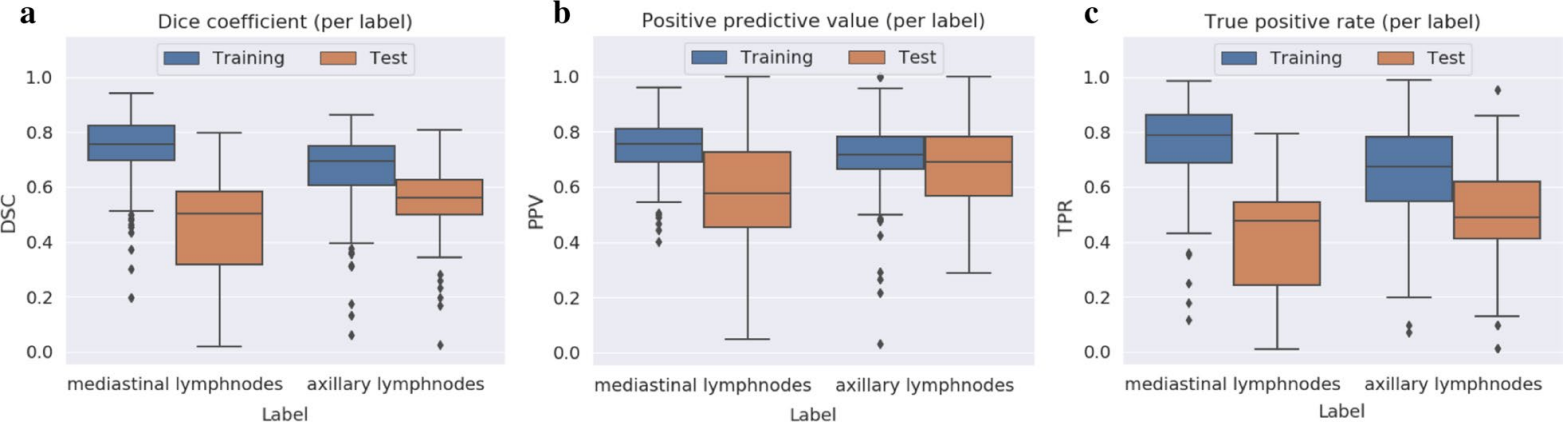
Overview of dice (**a**), positive predictive value (**b**) and true positive rate (**c**) training and validation data for mediastinal and axillary lymph nodes

Overall, a mean Dice value of 0.75 and 0.48 is achieved on the training and validation dataset. True positive rate and positive predictive value account to 0.76 and 0.75 on the training and 0.45 and 0.62 on the validation data. The Dice value for the mediastinal LN accounts to 0.44 and to 0.55 for the axillary LN with a smaller gap between training and validation, therefore showing less overfitting. More details can be seen in Fig. 3.

#### Lymph node detection rate according to lymph node size

**Fig. 4:**
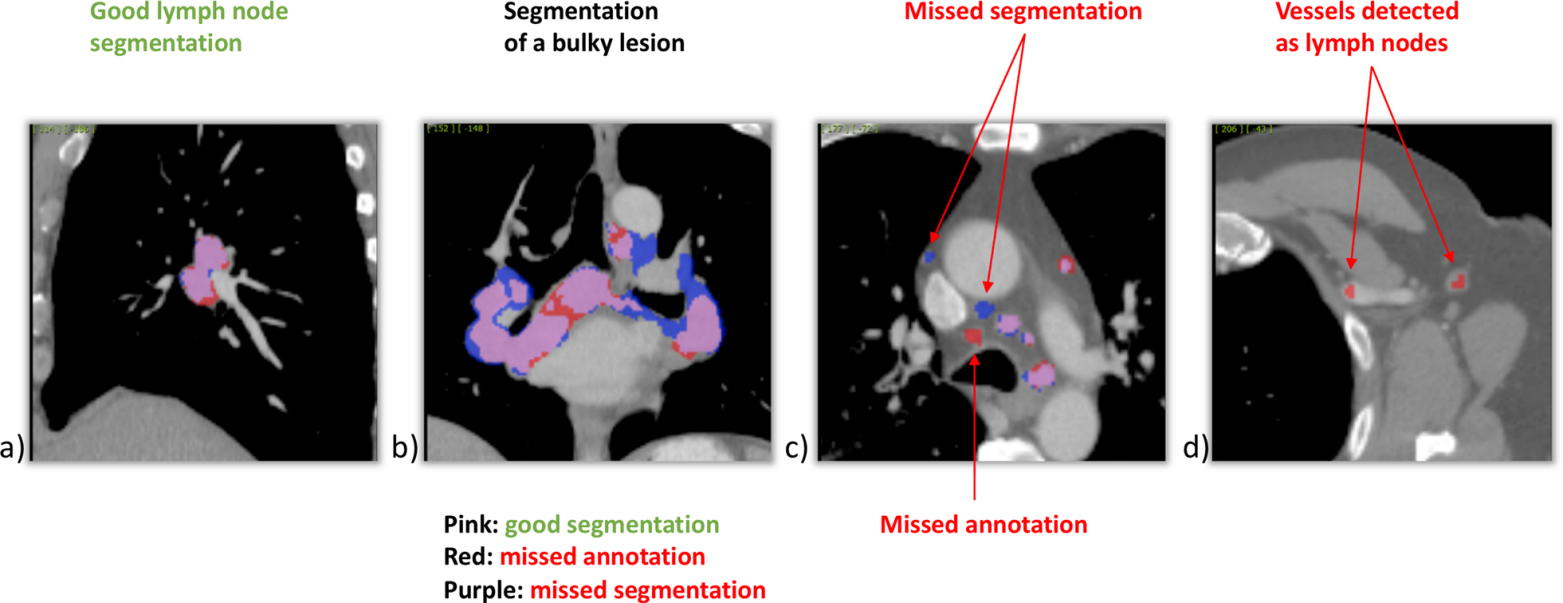
Examples of ground-truth and predicted segmentations. **a** Optimal LN segmentation, **b** segmentation of a LN bulk, **c** purple—missed LNs; red—true positive, detected LN which was initially not segmented by the radiologist (short-axis diameter < 5 mm); **d** red—false positive segmentation (vessels detected as LN). *LN* lymph node

**Fig. 5:**
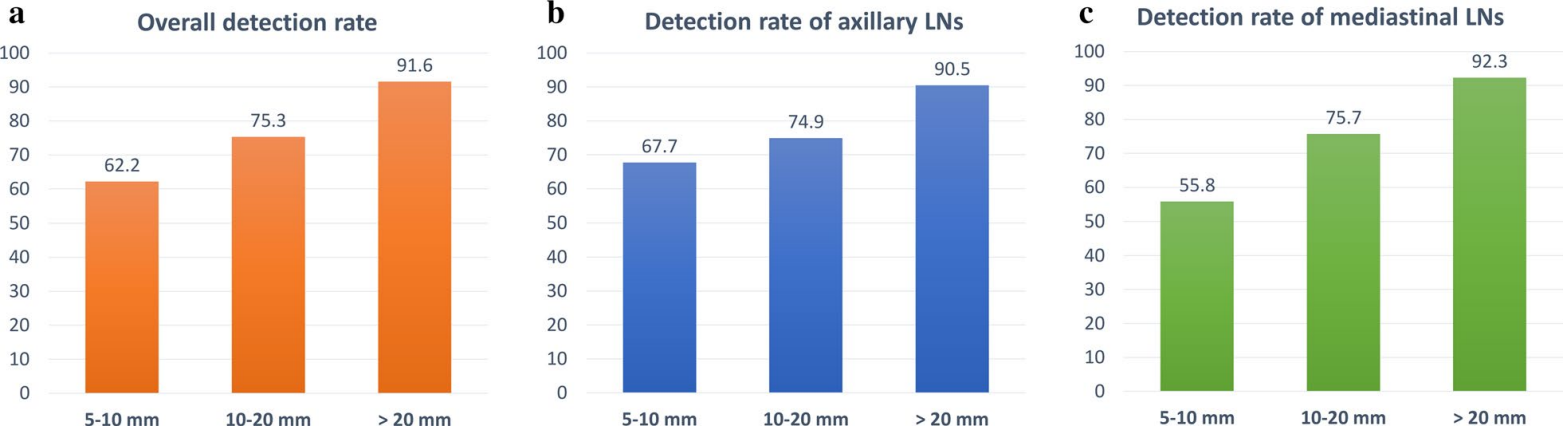
Overview of the validation detection rates depending on the short-axis diameter of the segmented LNs: 5–10 mm (2523 LNs), 10–20 mm (954 LNs), and > 20 mm (107 LNs). **a** Overall detection rates of both axillary, mediastinal and hilar LNs; **b** detection rates of axillary LNs, **c** detection rates of mediastinal and hilar LNs. *LNs* lymph nodes

The overall detection rate for all LNs with an SAD > 5 mm using the trained network was 66.5% with 10.3 FPs per volume on average. Exemplary images of detected and missed LNs compared to the ground truth segmentations are shown in Fig. 4. The highest detection rate could be observed when looking only at LNs with an SAD > 20 mm, while detection rate was only good to moderate when considering smaller LNs (SAD > 20 mm vs. SAD 10–20 mm: 91.6% vs. 75.3%, *p* < 0.001; SAD > 20 mm vs. SAD 5–10 mm: 91.6% vs. 62.2%, *p* < 0.001; Fig. 5). Looking only at the subgroup of clinically relevant enlarged LNs (defined by an SAD > 10 mm), a total detection rate of 76.9% was obtained with a significantly higher detection rate for LNs with an SAD > 10 mm as compared to LNs with an SAD < 10 mm (76.9% vs. 62.1%, *p* < 0.001; Fig. 5).

#### Lymph node detection rate according to lymph node location

A better overall detection rate was obtained for the axillary LNs compared to mediastinal LNs (70.0% vs. 62.3%, *p* < 0.001; Fig. 5). A better detection could be observed when looking only at LNs with an SAD > 20 mm, while detection rate was only good to moderate when considering smaller LNs, both for axillary and mediastinal LNs; axillary LNs with an SAD > 20 mm versus SAD 10–20 mm: 90.5% versus 74.9%, *p* < 0.001; SAD > 20 mm versus SAD 5–10 mm: 90.5% versus 47.2%, *p* < 0.001; Fig. 5); mediastinal LNs with an SAD > 20 mm versus SAD 10–20 mm: 92.3% versus 75.7%, *p* < 0.001; SAD > 20 mm versus SAD 5–10 mm: 92.3% versus 33.8%, *p* < 0.001; Fig. 5). Looking only at the subgroup of clinically relevant enlarged LNs (defined by an SAD > 10 mm), a slightly better detection rate was shown for LNs of the mediastinal region compared to the axillary (77.8% vs. 76.0%, *p* < 0.05).

**Fig. 6:**
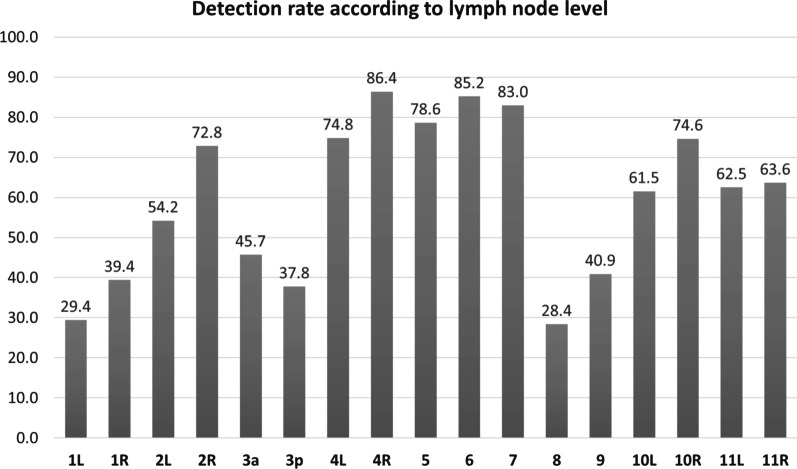
Overview of the validation detection rates of the mediastinal and hilar lymph nodes according to the localization of the lymph nodes. *R* right, *L* left

Based on the labelling of the mediastinal LNs a further analysis was performed to establish detection rates at different levels (Fig. 6). The best detection rates were obtained for LNs located in Level 4R (83.6%), and Level 7 (80.4%), while the lowest detection rate was recorded for LNs located in Level 8 (25.9%). A better detection rate was shown for LNs > 10 mm for all levels. For example, level 2 R (right) showed a detection rate of 96.5% for LNs > 10 mm versus 63.5% for LNs < 10 mm. For level 7, a total detection rate of 93.3% was shown for LNs > 10 mm versus 72.0% for LNs < 10 mm. The detection rate was statistically significant different for different levels (Table 2).

**Table 2:** Comparison matrix for *p* values of systematic differences in validation detection rates between lymph node levels

p VALUES	1L	1R	2L	2R	3A	3P	4L	4R	5	6	7	8	9	10L	10R	11L	11R
1L		0.29	**0.02**	**< 0.001**	0.08	0.4	**< 0.001**	**< 0.001**	**< 0.001**	**< 0.001**	**< 0.001**	0.96	0.32	**0.02**	**< 0.001**	**0.02**	0.04
1R	0.29		0.34	**< 0.001**	0.75	0.76	**< 0.001**	**< 0.001**	**< 0.001**	**< 0.001**	**< 0.001**	0.21	0.92	0.17	**< 0.001**	0.11	0.15
2L	**0.02**	**0.34**		**< 0.001**	0.28	0.14	**< 0.001**	**< 0.001**	**< 0.001**	**< 0.001**	**< 0.001**	**< 0.001**	0.5	0.45	**< 0.001**	0.26	0.32
2R	**< 0.001**	**< 0.001**	**< 0.001**		**< 0.001**	**< 0.001**	0.83	**0.01**	0.75	0.97	0.08	**< 0.001**	**0.01**	0.16	0.5	0.57	0.69
3A	0.08	0.75	0.28	**< 0.001**		0.42	**< 0.001**	**< 0.001**	**< 0.001**	**< 0.001**	**< 0.001**	**0.01**	0.9	0.15	**< 0.001**	0.11	0.17
3P	0.4	0.76	0.14	**< 0.001**	0.42		**< 0.001**	**< 0.001**	**< 0.001**	**< 0.001**	0	0.29	0.72	0.08	**< 0.001**	0.06	0.1
4L	**< 0.001**	**< 0.001**	**< 0.001**	0.83	**< 0.001**	**< 0.001**		**< 0.001**	0.9	0.89	0.1	**< 0.001**	**0.01**	0.12	0.59	0.51	0.64
4R	**< 0.001**	**< 0.001**	**< 0.001**	**0.01**	**< 0.001**	**< 0.001**	**< 0.001**		**0.01**	**0.02**	0.51	**< 0.001**	**< 0.001**	**0.01**	0.12	0.12	0.22
5	**< 0.001**	**< 0.001**	**< 0.001**	0.75	**< 0.001**	**< 0.001**	0.9	**0.01**		0.81	0.13	**< 0.001**	**0.01**	0.11	0.68	0.48	0.61
6	**< 0.001**	**< 0.001**	**< 0.001**	0.97	**< 0.001**	**< 0.001**	0.89	**0.02**	0.81		0.13	**< 0.001**	**0.01**	0.16	0.56	0.57	0.68
7	**< 0.001**	**< 0.001**	**< 0.001**	0.08	**< 0.001**	**< 0.001**	0.1	0.51	0.13	0.13		**< 0.001**	**< 0.001**	**0.02**	0.39	0.19	0.31
8	0.96	0.21	**< 0.001**	**< 0.001**	**0.01**	0.29	**< 0.001**	**< 0.001**	**< 0.001**	**< 0.001**	**< 0.001**		0.26	**0.01**	**< 0.001**	**0.01**	0.04
9	0.32	0.92	0.5	**0.01**	0.9	0.72	**0.01**	**< 0.001**	**0.01**	**0.01**	**< 0.001**	0.26		0.26	**< 0.001**	0.16	0.2
10L	**0.02**	0.17	0.45	0.16	0.15	0.08	0.12	**0.01**	0.11	0.16	**0.02**	**0.01**	0.26		0.08	0.64	0.64
10R	**< 0.001**	**< 0.001**	**< 0.001**	0.5	**< 0.001**	**< 0.001**	0.59	0.12	0.68	0.56	0.39	0	**< 0.001**	0.08		0.38	0.51
11L	**0.02**	0.11	0.26	0.57	0.11	0.06	0.51	0.12	0.48	0.57	0.19	**0.01**	0.16	0.64	0.38		0.95
11R	0.04	0.15	0.32	0.69	0.17	0.1	0.64	0.22	0.61	0.68	0.31	0.04	0.2	0.64	0.51	0.95	

*R* right, *L* left. Note that statistical significant *p* values are marked in bold.

### Network performance: testing dataset

On our in-house dataset, which was unseen during training, a detection rate of 69.9% was achieved for the enlarged LNs (SAD > 10 mm). This result compares well to the 76.9% achieved on the validation data set. It shows the generalization capabilities of our network which is able to cope with the domain shift when applied to images with a different pathology (bronchial cancer in the testing data, unclear cancer in the training and validation data).

## Discussion

The aim of the study was to develop a 3D DL algorithm for robust LN detection and segmentation in contrast-enhanced CT scans of the thorax. The main findings can be summarized as follows: (1) The algorithm achieved a good overall performance with an overall validation detection rate of 70% for LNs with an SAD over 5 mm. (2) Reasonable generalizability was achieved with a similar detection rate for enlarged LNs (SAD > 10 mm) in the fourfold cross-validation dataset compared to the unseen testing dataset of 76.9% and 69.9%, respectively. (3) A better validation detection rate was observed for enlarged LNs compared to smaller LNs (enlarged LNs showed a detection rate of 76.9%; the detection rate for LNs with an SAD ≥ 20 mm and SAD 0–5 mm was 91.6% and 40.8%, respectively). (4). Regarding different LN locations, the best validation detection rates were obtained for LNs located in Level 4R (right mediastinal), Level 7 (mediastinal subcarinal), and Level 10 R (right hilar) of 83.6%, 80.4% and 74.6%, respectively. (5) Segmentation accuracy shows a promising Dice value of 0.48. Segmentation accuracy is superior in the axillary region with less overfitting. This is probably due to the stronger homogeneity of the data compared to the mediastinal LNs.

Although a few DL approaches have been proposed for mediastinal LNs [21, 22, 26], there is still only a very limited number of publications available. A study similar to this work using the same evaluation criteria, employs a 3D u-net with additional organ segmentation masks as input for mediastinal LN segmentation [26]. A detection rate of 95.5% is reported on a different dataset including considerably fewer cases, thus impeding comparison to this study. The current approach did not rely on explicit shape modeling nor did it incorporate segmentation of neighboring organs. In addition, both axillary and mediastinal regions were simultaneously addressed, thereby providing a complete assessment of the thoracic region. Moreover, in contrast to other publications a total of 3585 LNs have been used for the training dataset only.

Previous studies using the same public dataset reported detections rates of 78% [21], 84% [22] and up to 88% [27] with 6 FPs per scan. In those studies, only the center of the LN was detected, and a detection was counted as correct if the detected landmark was within a distance of 15 mm from the ground truth landmark annotation. These detection rates are in good agreement with the validation results of this study while the current approach simultaneously provides a 3D segmentation of the LNs. Therefore, the algorithm can ensure a correct whole-volume segmentation of small as well as large LNs, necessary for the extraction of radiomic features in future approaches. Further, the whole-volume assessment of the network should potentially facilitate future work considering automated determination of total tumor load at diagnosis and in treatment response evaluation.

In contrast to previous studies, where only cross-validation (sixfold [21, 27] and threefold [22]) was performed, additional testing has been performed on a completely independent previously unseen dataset in addition to the fourfold cross-validation, in order to assess the generalizability of the trained network. Testing showed a similar detection rate compared to the initial fourfold cross-validation dataset, thus achieving a reasonable generalizability and facilitating LN detection during routine clinical work.

This work considered both axillary and mediastinal LNs using a single convolutional neural network, showing good validation results while addressing two different anatomical regions and therefore offering a complete analysis of the entire thorax with only one network.

Another way to potentially improve the detection rate is by increasing the amount of training data. Multiple, stronger data augmentation strategies, which have not been explored in the present study, have been proposed to improve vision tasks for images [28, 29].

CT scans containing bulky axillary or mediastinal lymphadenopathy have not been excluded. Even if the delimitation and segmentation of individual LNs forming the lesions was challenging, consecutively influencing the overall detection ICH rate negatively, these CT scans ensure a heterogeneous dataset.

The analysis by location showed considerable differences for the different LN levels. For example, for Level 4 LNs a validation detection rate of 85.0% was achieved for those localized on the right side whereas of only 71.0% for those on the left side. A possible explanation could be the considerable difference in the number of annotated LNs—262 LNs in level 4R and only 160 LNs in level 4L. A similar difference could be observed for Level 10 (75.0% with 71 annotated LNs for the right side versus 55.0% with only 29 annotated LNs for the left side). Additionally, worse contrast to surrounding tissue on the left versus right side might be another reason for the differences in detection rates.

Proper LNs classification and labelling is needed in order to develop future approaches in the characterization of malignant LNs, for example when considering Radiomics. Moreover, other features regarding the morphology of the thoracic LNs in addition to size (for example shape or homogeneity) should also be considered in future work. The current work considers just the thoracic LNs. Future work will address the extension to the abdominal region.

The main limitation of this study was the fact that the datasets were segmented by only one radiologist. However, this radiologist was well trained in detection and segmentation of LNs in chest CTs (more than 4 years of experience) and unclear LNs were discussed with an experienced radiologist (more than 15 years of experience). We assumed to have a homogenous dataset of the more than 4.000 manually segmented LNs with optimized inter-rater variability. Nevertheless, in this study the inter-rater effect of independent segmentation datasets for training of the network has not been evaluated. This was beyond the purpose of this study and has to be investigated in a subsequent trail.

Another limitation of the study is the limited number of annotated LNs. Adding more annotations to the training dataset could most probably ensure a better detection rate, especially for the mediastinal LNs located in levels for which the analyzed dataset had just few representatives.

Finally, another limitation of the study is the limited number of data augmentation strategies that has been applied, since multiple and stronger strategies could also potentially improve the detection rate.

## Conclusions

In conclusion, based on extensive and rigorous annotations, the proposed 3D DL approach achieved a good performance in the automatic detection and segmentation especially of enlarged LNs. In contrast to other work, both the axillary and mediastinal regions have been simultaneously addressed and thus a complete assessment of the thoracic region is provided. Our approach could be considered for further research regarding quantitative features of LNs to improve and accelerate diagnosis. Extension to other regions should be considered in the future.

## Data Availability

The training and validation dataset supporting the conclusions of this article is available at https://wiki.cancerimagingarchive.net/display/Public/CT+Lymph+Nodes or through request to the corresponding author. The testing dataset supporting the conclusions of this article can be accessed through request to the corresponding author.

## Abbreviations

AI: Artificial intelligence
BN-L: Batch normalization layer
CBR: Convolutional layer (Conv-L), batch normalization layer (BN-L) and ReLU layer (ReLU-L)
CBRU: Convolutional layer (Conv-L), batch normalization layer (BN-L) and ReLU layer (ReLU-L), followed by upsampling layers
CNTK: Microsoft Cognitive Toolkit
Conv-L: Convolutional layer
CT: Computed tomography
DL: Deep learning
f-net: Foveal neural network
FP: False positive
GPU: Graphics processing unit
HU: Hounsfield units
LN: Lymph node
RECIL: Response evaluation criteria in lymphoma
RECIST: Response evaluation criteria in solid tumors
ReLU-L: Rectified linear units layer
SAD: Short-axis diameter
u-net: Fully convolutional networks
